# The hidden crisis: Long COVID’s association with housing stability and home accessibility among people with disabilities

**DOI:** 10.1016/j.dhjo.2024.101650

**Published:** 2024-06-07

**Authors:** Kelsey Goddard, Andrew Myers, Catherine Ipsen

**Affiliations:** a University of Kansas, Institute for Health and Disability Policy Studies (KU-IHDPS), 1000 Sunnyside Ave., Room 1052, Lawrence, KS, 66045, USA; b University of Montana, Research and Training Center on Disability in Rural Communities (RTC:Rural), 253 Corbin Hall, Missoula, MT, 59812, USA

**Keywords:** 3–5): long COVID, COVID-19 pandemic, Housing stability, Home accessibility

## Abstract

**Background:**

The COVID-19 pandemic has given rise to the emerging phenomenon known as Long COVID, characterized by persistent symptoms long after the acute infection has passed. However, the relationship of Long COVID on housing stability and home accessibility remains underexplored.

**Objective:**

This manuscript aims to comprehensively examine the association of Long COVID on housing stability and accessibility, identifying challenges faced by people with Long COVID and potential strategies to address them.

**Methods:**

The study employs a cross-sectional mixed-methods approach, combining quantitative and qualitative methods. It analyzes data from 1533 people with disabilities, 514 with Long COVID and 1019 without Long COVID, to compare demographics, housing stability, financial concerns, housing problems, and home accessibility. Qualitative analysis extracts thematic insights from 13 participant narratives.

**Results:**

Individuals with Long COVID exhibit significantly higher rates of housing instability (21.1 % v. 8.1 %, p < 0.001) and financial concerns, such as worries about high rent or mortgage (50.4 % v. 40.0 %, p < 0.001), compared to those without Long COVID. They also report more frequent issues with pests (30.0 % v. 23.5 %, p < 0.01) and mold (22.0 % v. 12.7 %, p < 0.001) in their homes. Qualitative analysis reveals financial setbacks, difficulties in obtaining support, and the challenges of home accessibility.

**Conclusions:**

Associations between Long COVID and challenges related to housing stability and home accessibility highlight the need for systemic changes, financial support, and advocacy. This research contributes to understanding Long COVID’s challenges, informing policy development, and promoting compassionate responses, ensuring the well-being of people with Long COVID.

## Introduction

In the wake of the COVID-19 pandemic, our understanding of the virus’s far-reaching consequences continues to evolve. Despite advancements in COVID-19 diagnosis, treatment, and prevention,^1–3^ the emergence of Long COVID introduces a complex and multifaceted challenge to many disabled people who already face significant housing barriers.^4,5^ The Centers for Disease Control and Prevention (CDC) notes a significant prevalence, with 1 in 13 U.S. adults (7.5 %) now grappling with this condition, defined as symptoms lasting 3 months or more after the initial virus contraction.^6^ Amid this growing understanding, one area that remains largely unexplored in the context of Long COVID is its potential impact on housing stability and home accessibility, specifically for people with disabilities.

The persistent and varied symptoms, including debilitating fatigue, cognitive impairment, and shortness of breath,^3^ have the potential to disrupt an individual’s ability to maintain stable housing and navigate their home environment effectively.^7,8^ These symptoms can significantly impair daily functioning, making it challenging to manage employment^9^ and fulfill financial obligations,^10^ thereby heightening the risk of eviction, foreclosure, or failing to meet the ongoing demands of home maintenance.^7^ Long COVID can exacerbate existing disabilities or introduce new ones,^11^ necessitating adaptations in the living space ranging from minor modifications (e.g., grab bars) to more significant accommodations (e.g., wheelchair ramps, specialized room layouts), or even necessitate relocation to a more suitable living environment.^12^

For individuals with disabilities, stable housing is not merely a place to live but a critical factor in the ability to manage health conditions, maintain independence, and engage in community life. Research shows that maintaining housing stability, which encompasses the ability to stay in one’s home without the threat of eviction or foreclosure, and having a home that is accessible and accommodating is essential for overall well-being and recovery.^13,14^ In this context, housing stability not only refers to the physical suitability of the living environment but also its financial affordability, ensuring that individuals can maintain their homes over the long term without the threat of displacement. Therefore, addressing the housing needs of individuals with Long COVID, particularly those with disabilities, requires a nuanced understanding of the condition’s impact on the essential role of adaptive, stable housing.

While existing literature extensively covers the immediate impacts of the COVID-19 pandemic on housing stability,^15–18^ a gap exists in understanding Long COVID’s influence on such critical, long-term aspects of people’s lives. This manuscript aims to address this knowledge gap through comparative and qualitative analyses, examining the relationships and experiences related to housing stability and home accessibility among individuals with Long COVID. We hypothesize that individuals with Long COVID experience greater housing instability and accessibility challenges compared to those without Long COVID, as evidenced by increased rates of financial concerns, housing-related problems, and the need for home adaptations to accommodate new or exacerbated disabilities. Utilizing both quantitative and qualitative data, we delve into risk factors, potential interventions, and policy recommendations to mitigate these challenges, contributing valuable insights for public health responses and policy development within the broader context of Long COVID.

## Methods

### Data source and sample

This cross-sectional study utilized unweighted data from the 2022 National Survey on Health and Disability (NSHD), an internet-based survey of people with disabilities in the United States, conducted from May to September of 2022. Given the inherent challenges in defining and enumerating a representative population of individuals with disabilities—stemming from the limitations of federal question sets which do not fully capture this demographic^19,20^—the NSHD adopted a purposive convenience sampling method. This approach aimed to engage a broad and more inclusive array of respondents.

The target population for the NSHD consists of adults residing in the United States who self-identify as having *“any physical or mental condition, impairment, or disability that affects daily activities and/or requires the use of special equipment/devices.”* This disability screening measure aims to capture a wide spectrum of experiences and conditions often over-looked by more restrictive federal definitions.

The recruitment process involved collaboration with over 60 national disability organizations, leveraging their networks to distribute the survey broadly. These organizations utilized various communication channels, including emails, newsletters, and social media, to reach potential respondents, thereby enhancing the survey’s visibility and reach within the disability community.

The survey garnered responses from 2725 adults with disabilities, reflecting a broad array of experiences and conditions. However, due to the limitations in federal question sets, we cannot confirm the representativeness of our sample within the broader disability population. This uncertainty underscores the complexity of defining a representative sample in this context. Additionally, because we used a convenience sampling approach, we do not have data on the survey’s response rates. Participants were incentivized with a drawing for one of ten $100 gift cards. All study procedures were approved by the University of Kansas Institutional Review Board.

### Quantitative measures

#### Long COVID status.

Respondents were classified into “Long COVID” (*n* = 514) and “non-Long COVID” (*n* = 1019) groups based on their response to the question, *“Have you experienced any COVID-19 symptoms for three months or longer following a suspected or confirmed COVID-19 infection* – *also known as Long COVID? Examples of symptoms include fatigue or extreme tiredness, cognitive problems, abnormal heart rate, shortness of breath, loss of taste or smell, depression or other mental health conditions.”* Due to the survey design, participants who did not confirm having contracted COVID-19 or responded *“I don’t know”* were not asked the Long COVID question, leading to the exclusion of 1192 respondents.

#### Sociodemographic characteristics.

Information collected included housing status [own, rent, live long-term with friends or family, and other situation], living situation [alone or with others], gender, race, ethnicity, age, self-reported health, and employment status.

#### Housing variables.

“Housing instability,” adapted from the Social Needs Screening Tool,^21^ was categorized based on participants’ responses to their current living situation. Individuals who reported a lack of a steady place to live or concerns about losing their place of residence within the next six months were identified as having housing instability. “Housing cost burdens,” adapted from the American Housing Survey (AHS),^22^ encompassed perceptions of high rent or mortgage payments and real estate taxes, with “high” being a subjective term based on participants’ own assessments rather than a predefined threshold. This category also included utility bills, unsteady income, reliance on others for housing expenses, unexpected expenses, and high debt, each scored as either present (1) or absent (0). “Housing-related problems,” adapted from the Social Needs Screening Tool,^21^ included pests, mold, lead paint or pipes, lack of heat, malfunctioning appliances, missing or non-functioning smoke detectors, and water leaks, recorded as *“yes”* (1) if present. “Housing accessibility,” an internally developed measure, was assessed based on the question, *“How well do you think the layout and features of the place you live now* support *you?”*, rated from *“not at all”* (1) to *“very well”* (5). Quantitative data analyses were conducted using cross-tabulations in SPSS (Version 28). Differences between the Long COVID and non-Long COVID groups were tested using chi-square tests.

### Qualitative methods

We focused exclusively on individuals with Long COVID and conducted 13 in-depth remote interviews using Zoom and telephone, drawing from a purposively selected sample of NSHD respondents. This sample was carefully chosen to represent a diverse range of demographics, including housing status, gender, race, ethnicity, and geographical region. Our semi-structured interview guide, refined through pilot interviews and expert feedback, was developed to ensure comprehensive coverage of relevant themes, such as the participants’ employment status, financial situations, and home environment interactions. During the interviews, the flexibility of our approach allowed for thorough exploration and spontaneous follow-up questions, enriching the data collected. Each interview was recorded and transcribed verbatim to maintain the integrity and accuracy of the participants’ responses, and participants received a $50 gift card for their contributions. The interviews were conducted from January to October 2023. Following the interviews, a single coder performed an initial thematic analysis on the transcripts,^23^ identifying potential themes and categorizing the data. These initial themes were then meticulously refined in relation to the data set and existing literature. To ensure the robustness of our findings, two additional members of the research team reviewed the initial coding. This collaborative effort aimed to validate and refine the identified themes, ensuring themes accurately reflected the participants’ experiences with Long COVID.

## Results

### Demographics

Table 1 compares demographics of individuals with Long COVID (*n* = 514) to those without (*n* = 1019). The Long COVID group had significantly more females (p < 0.01). Individuals with Long COVID were also significantly more likely to report poor/fair health and not working (p < 0.001). Demographics of interview participants are also included in Table 1.

### Quantitative findings

Table 2 presents a comparative analysis of housing stability and cost burdens between Long COVID and non-Long COVID groups. Housing instability was markedly higher among the Long COVID group (21.1 %) compared to non-Long COVID groups (8.1 %). Participants with Long COVID reported greater concerns about high rent or mortgage payments (50.4 % vs. 40.0 %), utility bills (55.7 % vs. 45.5 %), unsteady income (54.8 % vs. 35.2 %), relying on others for housing payments (46.6 % vs. 30.8 %), and large, unexpected expenses (80.3 % vs. 36.2 %). They also reported more concern about high credit card, student loan, or other debt (51.3 % vs. 38.1 %). However, concerns about high real estate taxes were similar, with 31.0 % of respondents in the Long COVID group and 27.1 % in the non-Long COVID group expressing concern. Regarding housing-quality issues, individuals with Long COVID reported more pest problems (30.0 % vs. 23.5 %) and more prevalent mold issues (22.0 % vs. 12.7 %). Other housing concerns showed minor differences. Concerning home accessibility, 17.6 % of individuals in the Long COVID group reported encountering unsupportive housing layouts and features, significantly higher than the 11.4 % reported among the non-Long COVID group.

### Qualitative findings

While Table 2 provides a comprehensive overview of housing stability and home accessibility among individuals with Long COVID, our qualitative investigation reveals the intricate ways in which Long COVID is associated with housing stability, financial burdens, housing issues, and home accessibility. Seven overarching themes, each accompanied by distinct key topics (shown in Table 3), offer unique insights into the challenges faced by these individuals. The subsequent narrative presents detailed descriptions and representative quotations for each theme, enhancing the depth of our findings.

### Direct impacts of long COVID

#### Theme 1: Employment and financial impact on housing stability.

The theme of employment and financial impact highlights the profound effects of Long COVID on participants’ ability to work. Changes to employment status, resulted in significant income loss and subsequent instability, which, in turn, affected housing stability. As participant #15724 states: *“I’ve been unable to work as a result [of Long COVID] … I was a geriatrician, a physician for old people. And I was very busy, never worked less than 40 h a week. I got infected at work and have been sick ever since. And that was three and a half years ago.”*

The issue of high-deductible health insurance plans adds another layer to the financial strain, as participant #15724 highlights: *“I was a healthy person going into this. I was a long-distance runner. I had no reason to think that I would really need my health insurance. So, I had a high deductible plan … For the final year I was on [my employer-sponsored insurance], I spent between $10,000 or $12,000.”*

The struggle to meet financial obligations extended beyond healthcare, affecting various aspects of participants’ lives such as bills, medications, and the pursuit of nutritious food. As participant #11561 illustrates: *“Aside from the little GoFundMe funds that I have gotten, which all went to bills, there’s no extra money for buying an electric wheelchair or getting a new car or finding a [more accessible] house … You just kind of feel stuck.”*

Participants also described the painful reality of depleting savings and liquidating assets like 401(k)s and personal belongings, a sentiment captured by participant #15340: *“For a while, I was paying my rent and the bare minimum expenses … just out of my savings account. I cashed in my 401k. I cashed in savings bonds that I’ve had since birth … So, I was just blowing through all the savings that I had amassed at this point in my life.”*

Finally, the necessity of lifestyle adjustments to cope with financial and health-related limitations is evident in participants’ experiences, as participant #15724 describes: *“I very much decreased my expenditures. We sold our house and moved to a much smaller place … Basically, we changed our lifestyle so that we could survive on just one income.”*

#### Theme 2: Home accessibility challenges.

Participants detailed significant difficulties with home accessibility, particularly with stairs, which became major obstacles in their daily lives due to physical limitations and fatigue from Long COVID. Participant #15340 provides a vivid account: *“The stairs are definitely difficult. They’re a lot of energy. Like do I have the energy to go up one more time … ? And it’s like no, I don’t. I’m down here. I’m done for the day. It just makes things a little bit more complicated … There’s no running water down here. So, if I need to wash my hands or get a get a drink, I’ve got to go upstairs … And I’m not able to hold my bladder as well as I used to. So, there was one or two accidents that I just cleaned up and don’t think about. Like everybody has an accident. It just sucks that I have to have them so young.”*

Kitchen accessibility was another critical issue, with many participants finding it challenging to prepare meals due to the lack of suitable modifications (e.g., lack of accessible countertops, cabinets, and appliances). As participant #15340 shares: *“I’ll get something on the stove to start boiling and then just come in the other room and sit down. And wait until I hear it start boiling because I can’t stand there. And I mean, I used to cook a lot … And now I just don’t have the energy.”*

Bathroom accessibility posed additional hurdles, with participants facing challenges using shower facilities, bathtubs, and the absence of assistive devices, significantly affecting their personal hygiene and safety. Participant #11561’s experience encapsulates these challenges: *“[Bathing] is so exhausting that I can only shower like once a week. And then it usually takes me about an hour and a half, or almost 2 h, just for the actual showering part, not even drying or getting dressed or doing anything. Because, at that point, I am laying on my bed recovering after. And I have to use bath wipes and a shower cap type of thing on the other days.”*

#### Theme 3: Effects of home inaccessibility on daily life.

Participants discussed the need to manage their energy carefully due to fatigue and physical limitations caused by Long COVID, necessitating prioritization of tasks to ensure well-being. As participant #15340 illustrates: *“I need to kind of gamble with my energy a lot of the times and what I can do. If my laundry doesn’t get done today, or if I need to finish it in the morning, that’s not that big of a deal. But I had to get the trash done today … I’m going to be on my bed the rest of today.”*

Inaccessible homes necessitated reliance on family members, friends, or caregivers for daily tasks and mobility within the home. Participants often found themselves unable to move around or use certain rooms without assistance, contributing to a heightened sense of dependence. As participant #11561 illustrates: *“There are about ten steps down to our driveway to get to one of the vehicles. I can’t get down them by myself. My husband has to help me … Or like if my sister or my aunt or somebody is taking me to an appointment, they help me down the stairs. And they have to bring the walker and the wheelchair down for me … I can’t do it alone.”*

The combination of inaccessible rooms and limited mobility raised the risk of injuries, particularly due to falls. Participants shared various instances of falls, broken bones, and accidents within their homes. As participant #11561 states: “*I don’t think I’ve reciprocal walked upstairs in at least a year and a half. It’s always a one step because I lose my balance. And, with the neuropathy, I can’t always tell if I’m on the step. I had four falls in a year when I first got sick. One of them resulting in a broken foot.”*

Inaccessibility within their homes prevented participants from engaging in community activities and events, leading to feelings of being trapped and isolated, exacerbating their sense of loneliness and exclusion. As participant #11561 notes: *“I physically can’t go outside of my house by myself. And it is isolating … My whole existence is in my house … And it’s literally all I have. My whole life is in this box. I live in a box.”*

One participant described a particularly challenging period when they became bed-bound, highlighting the critical impact of home inaccessibility on the risk of institutionalization. Participant #15642’s account is telling: *“I actually had considered and asked my health care professional if I needed to move into a [nursing] home because I was completely bed bound. I was in basically keeping bottles of Ensure around me just so I could drink it and not have to get up, and I was literally wearing a diaper because I lost all control of my muscles.”*

### Downstream consequences

#### Theme 4: Deteriorating housing conditions.

The theme of deteriorating housing conditions sheds light on the struggles many participants faced in maintaining their homes, a challenge directly linked to the physical limitations imposed by Long COVID. The inability to perform even minor repairs or routine upkeep due to debilitating symptoms was a common thread in participants’ experiences, highlighting a significant aspect of their daily lives disrupted by the illness. Participant #15724 vividly captured this sentiment: *“We’re constantly in a situation where we have little things that need to be done around the house. We don’t have the ability to do it, and we can’t find anyone to hire to do it … Things that are really, really little, but we’re just not capable of.”*

Compounding these challenges, participants also dealt with the breakdown of essential household appliances, adding layers of stress and practical difficulties to their already strained lives. As participant #15761’s experience exemplifies: “*Of course, my washing machine broke during all this … And not having the money to buy another one, I’m having to go to the laundromat … I may or may not get [my laundry] back in the house that same day. But it won’t get put away that same day … Lifting, moving, picking up, bending … I’ve got arthritis in every bone in my body now.”*

Moreover, some participants shared distressing accounts of how deteriorating housing conditions exacerbated their physical and emotional turmoil. As participant #14062 illustrates: *“The situation in my house that got so bad was they were doing some sewer repair outside. And it tore up the street. And all the rats in the neighborhood came above ground … And at this point, the dysautonomia was to such a degree that I would literally sometimes collapse. I could not stand up. I was laying in tarps because the diarrhea never stopped … And then I look up, and there’s rats climbing my curtains and coming over the picture molding above my bed…So now I have PTSD every night because this is what I picture when I’m falling asleep.”*

### Efforts to mitigate and barriers

#### Theme 5: Home modifications and daily living aids.

This theme explores how participants adapted their living environments to better manage the limitations imposed by Long COVID. Many participants undertook home modifications to enhance accessibility and support their daily activities (e.g., installing railings, shower chairs, and raised toilets), reflecting a proactive approach to mitigating the impact of their physical restrictions. As participant #15724 shares: *“My wife actually built a fold out countertop so I can pull up a stool and sit at the counter to do cooking or cutting things. Because, otherwise, if there’s not a countertop that sticks out, then my [legs] would not be able to go under the countertop, and I really wouldn’t be able to do it.”*

In addition to structural changes, participants incorporated various mobility aids (e.g., walkers and wheelchairs) to assist with navigation and reduce physical strain within their homes. As participant #15340 illustrates: *“I’ve got these little dolly cart things that I got from some furniture store. And I use those to get laundry or if I order a package…I use those to get from one end of the house to the other. And I’ve got one on each floor so that I can use them. It’s just easier on my body when I can just lean onto something and just push it.”*

The theme also touches on participants’ decisions to relocate to more suitable living spaces. Downsizing or choosing homes with accessibility in mind was a critical step for some, as participant #15724 illustrates: *“I had been in a colonial [house] where there was a long walk from my bedroom to the stairs to downstairs, and then another long walk to the kitchen. The setup was bad. And now I’m in a split level. I have only five steps and about 20 feet between me and the kitchen … So, that makes a really big difference that I don’t have to walk far to get to the kitchen or the bathroom.”*

#### Theme 6: Financial constraints on home modifications.

This theme underscores the financial hurdles participants encountered when considering necessary home modifications to accommodate their needs due to Long COVID. The cost of adapting homes for increased accessibility proved prohibitive for many, highlighting a significant gap between the need for such modification and the ability to afford them. Participant #11561’s statement encapsulates this challenge: *“We’ve tried to look into some different options for [home] modification. But it’s expensive. And we don’t have the finances to be able to really make any modifications.”*

Additionally, the theme touches of the difficulties of acquiring mobility aids, such as wheelchairs, which are crucial for maintaining independence and quality of life. As participant #15724 highlights: *“I looked into the process of getting a wheelchair and discovered that I had zero chance of it being approved by my insurance.”*

#### Theme 7: Lack of support from agencies and healthcare.

This theme delves into the challenges participants encountered while navigating support systems, emphasizing the difficulties in securing aid from both healthcare and social service agencies amidst the complexities of Long COVID. Participant experiences reveal a systemic struggle in accessing private and Social Security disability benefits, with bureaucratic hurdles significantly impeding their ability to obtain crucial financial support. As participant #15724 states: *“The system basically is designed to automatically deny everyone on first pass and force everyone to hire a lawyer … It wasn’t until a couple months before my administrative judge hearing that I heard from someone that there was a form called a residual functional capacity form … And it doesn’t appear that most doctors know anything about that.”*

The theme also captures participants’ efforts to seek state and private rental assistance, illustrating the temporary relief and subsequent challenges when such assistance runs its course. As participant #15340 reflects: *“Once [rental assistance] ran out, I wasn’t able to afford my rent anymore … So, I had to move. Right now, I am living in my grandparents’ basement because it’s rent free … Because I definitely can’t afford rent right now.”*

Moreover, the theme sheds light on the dissatisfaction and frustration with government agencies, particularly when it comes to healthcare and personal assistance services. Participant grievances about the inadequacies of Medicaid and other services illustrate the broader issue of unmet needs within this community. As participant #14062 notes: *“My situation here at the house became so bad that last year, I called Adult Protective Services … I called the suicide crisis hotline. And I asked the crisis line to report me as a victim of neglect. Because…No one. No one has come to help me.”*

## Discussion

People with pre-existing disabilities have long grappled with housing instability and accessibility concerns.^24^ These persistent housing problems^25^ are potentially exacerbated by Long COVID, emphasizing its role in a broader narrative of housing inequities.^26–28^ We should view the experiences of individuals with Long COVID as a catalyst for addressing broader issues and developing comprehensive solutions that benefit all individuals with disabilities. This study emphasizes the pressing need to address these long-standing concerns, both within the context of Long COVID and as part of a larger initiative to create inclusive and accessible housing for all.

Our findings indicate an association between individuals with Long COVID and experiences of housing instability, which may be associated with high costs and unsteady income. Healthcare practitioners should be aware of the financial challenges faced by these patients and consider referring them to financial counseling or support services, including Medicaid or other assistance programs, which can provide critical support in accessing various necessary services.^29^

Policy changes, such as extended rent and mortgage relief programs^30^ or enhanced job protection for individuals with prolonged COVID symptoms,^31^ are crucial to address these financial barriers. Additionally, systemic changes are needed to ensure economic stability for individuals with disabilities, ensuring they have access to affordable housing options. Tailored financial support, potentially accessible through Medicaid or specific grants designed for individuals with disabilities, could alleviate some of the economic pressures contributing to housing instability.

Furthermore, our study reveals that some people with Long COVID report struggling to maintain their homes due to physical limitations, impacting both housing stability and overall health. Healthcare providers should assess the living conditions^32^ and recommend necessary home health or rehabilitation services. Policymakers should also consider implementing incentives or programs to assist Long COVID patients in maintaining their living environments. In this regard, systemic changes could include policies that mandate or incentivize the construction of accessible housing units,^27^ ensuring that new developments meet the needs of individuals with varying levels of mobility and physical ability.

Our findings also highlight that some people with Long COVID encounter home accessibility challenges, which may significantly affect their energy levels, reduce their independence, and increase their risk of injuries. To effectively address these challenges, collaborative efforts between healthcare practitioners, occupational therapists, and physical therapists are essential.^33^ These professionals can provide a holistic approach to care, ensuring that the individual’s living environment is conducive to their recovery and daily functioning. Healthcare providers also play a critical role in assisting patients in navigating resources for adaptive equipment and home modifications,^34^ with potential support from Medicaid or similar programs to facilitate these adaptations. This support from healthcare providers is vital in supporting individuals with Long COVID adapt to their new limitations, promoting safety and autonomy within their living spaces.

On a broader scale, policymakers must recognize the importance of supporting home adaptations for individuals with Long COVID by expanding insurance coverage and grant programs, including Medicaid. Such initiatives would not only provide financial relief but also ensure that more people have access to the necessary modifications that can significantly improve their quality of life. By fostering an environment where comprehensive support is readily available, we can make a meaningful difference in the lives of those affected by Long COVID, helping them navigate their challenges more effectively.

## Limitations

One limitation is the potential bias in self-reported data, which might affect the study’s accuracy. The cross-sectional study design precludes causal inferences and examination of changes over time. Qualitative data, while valuable, may not capture the full diversity of Long COVID experiences, and subjectivity in qualitative data analysis may lead to different interpretations. Additionally, the researchers did not inquire whether respondents were currently experiencing Long COVID symptoms, which limits our understanding of the impacts of Long COVID on housing.

Moreover, the study’s findings could be influenced by various confounding factors that were not controlled for, such as preexisting health conditions, socioeconomic status, and access to healthcare services. For instance, preexisting health conditions might exacerbate the severity of Long COVID symptoms, thereby increasing the individual’s housing instability or need for accessible housing. Therefore, future research should consider these potential confounding factors to provide a more nuanced understanding of the observed relationships. Finally, the study did not extensively explore regional or contextual factors influencing housing stability and accessibility, which could offer additional insights into the barriers and facilitators of housing equity for people with Long COVID.

Additionally, we acknowledge the inherent limitations of making inferences from nonprobability samples to the broader population. While our sample may not be representative of the general population, it is nevertheless fit for the purpose of this study, which involves examining relationships between population characteristics and key outcomes rather than focusing on the prevalence of those outcomes. This approach allows us to explore the complex dynamics and specific challenges faced by individuals with Long COVID in relation to housing stability and accessibility.

## Conclusion

This manuscript underscores the profound connection between Long COVID and issues with housing stability and accessibility, which persist beyond the initial infection. Individuals with Long COVID may grapple with heightened rates of housing instability, financial stress, and home accessibility challenges. Implementing systemic changes and tailored financial support is crucial to enhance living conditions and alleviate the shortage of accessible housing. This research contributes to a broader understanding of Long COVID challenges and advocates for a more inclusive response to the pandemic’s aftermath. We hope our findings spur further investigations, shape policy development, and promote a compassionate, evidence-based approach to support individuals with Long COVID.

## Figures and Tables

**Table 1:** Participant demographics.

	Long COVID (*n* = 514)	Non-Long COVID (*n* = 1019)	Interview Participants (*n* = 13)
Housing Status			
Own	46.4 %	46.8 %	*n* = 6
Rent	36.9 %	37.6 %	*n* = 5
Long-term w/friends/family (not paying rent)	13.1 %	13.0 %	N/A
Other Situation	3.6 %	2.6 %	*n* = 2
Lives Alone	23.5 %	24.6 %	*n* = 6
Gender**			
Female	70.8 %	63.6 %	*n* = 9
Male	17.4 %	25.5 %	N/A
Transgender	0.2 %	0.4 %	N/A
Non-Binary	6.1 %	4.5 %	*n* = 2
Two-Spirit	0.8 %	0.1 %	*n* = 1
Agender	1.2 %	1.8 %	N/A
Other^a^	3.6 %	4.1 %	*n* = 1
Race			
American Indian/Alaska Native	1.9 %	1.1 %	*n* = 3
African American	3.1 %	3.3 %	*n* = 2
Asian	2.3 %	2.8 %	*n* = 1
Native Hawaiian/Pacific Islander	0.0 %	0.1 %	N/A
White	87.6 %	88.9 %	*n* = 5
Multi-Racial	5.2 %	3.7 %	*n* = 2
Ethnicity			
Hispanic/Latine	7.0 %	5.6 %	*n* = 1
Poor/Fair Health***	68.6 %	40.4 %	*n* = 10
Employment Status***			
Not Working	38.4 %	26.5 %	*n* = 7
Working for Pay	42.6 %	49.2 %	*n* = 5
Self-Employed	8.4 %	7.7 %	N/A
Both Self-Employed & Working for Pay	3.9 %	4.2 %	N/A
Not Working, Retired	6.6 %	12.4 %	*n* = 1
Geographical Region			
South	27.2 %	24.5 %	*n* = 3
West	28.4 %	28.8 %	*n* = 3
Midwest	26.3 %	24.3 %	*n* = 3
Northeast	18.1 %	22.2 %	*n* = 4
Territories	0.0 %	0.2 %	N/A
Mean Age*	44.8 (18–79; SD = 12.7)	45.8 (18–92; SD = 15.0)	43.7 (23–63; SD = 12.5)

* *p* < 0.05;

** *p* < 0.01;

*** *p* < 0.001.

a Includes other genders as identified by participants.

**Table 2:** Comparative analyses.

	Long COVID (*n* = 514)	Non-Long COVID (*n* = 1019)
Housing stability and cost burdens		
Experiencing Housing Instability***	21.1 %	8.1 %
Worried About High Rent or Mortgage***	50.4 %	40.0 %
Worried About High Utility Bills***	55.7 %	45.5 %
Worried About Unsteady Income***	54.8 %	35.2 %
Worried About Relying on Others to Help Cover Housing Payments***	46.6 %	30.8 %
Worried About Large Unexpected Expenses***	80.3 %	36.2 %
Worried About High Credit Card, Student Loan, or Other Debt***	51.3 %	38.1 %
Worried About High Real Estate Taxes	31.0 %	27.1 %
**Housing-quality problems**		
Problems with Pests**	30.0 %	23.5 %
Problems with Mold***	22.0 %	12.7 %
Problems with Lead Paint/Pipes	3.7 %	3.1 %
Problems with Lack of Heat	3.7 %	2.6 %
Problems with Oven, Stove, or Refrigerator Not Working	7.6 %	5.5 %
Problems with Smoke/CO2 Detectors Missing or Not Working	10.9 %	9.0 %
Problems with Water Leaks	11.3 %	9.7 %
**Housing accessibility**		
Unsupportive Housing Layout and Features***	17.6 %	11.4 %

* *p* < 0.05;

** *p* < 0.01;

*** *p* < 0.001.

**Table 3:** Qualitative themes and key topics.

**Theme 1: Employment and Financial Impact on Housing Stability**
Loss of Work and Income
Health Insurance Costs
Monthly Financial Struggles
Depletion of Savings and Retirement Funds
Financial Adjustments
**Theme 2: Home Accessibility Challenges**
Stair Challenges
Kitchen Accessibility
Bathroom Limitations
**Theme 3: Effects of Home Inaccessibility on Daily Life**
Decreased Energy
Restricted Independence
Increased Risk of Injury
Decreased Community Participation/Isolation
Increased Risk for Institutionalization
**Theme 4: Deteriorating Housing Conditions**
Maintenance Challenges
Appliance Breakdown
Traumatic Experience
**Theme 5: Home Modifications and Daily Living Aids**
Home Modifications
Use of Mobility Aids
Housing Relocation
**Theme 6: Financial Constraints on Home Modifications**
Financial Constraints on Home Adaptations
Financial Constraints on Obtaining Mobility Aids
**Theme 7: Lack of Support from Agencies and Healthcare**
Barriers in Obtaining Financial Support and Disability Benefits
Barriers in Maintaining Rental Assistance
Absence of Assistance
